# Comparative analysis of volatile metabolomics signals from melanoma and benign skin: a pilot study

**DOI:** 10.1007/s11306-013-0523-z

**Published:** 2013-03-30

**Authors:** T. Abaffy, M. G. Möller, D. D. Riemer, C. Milikowski, R. A. DeFazio

**Affiliations:** 1 Molecular and Cellular Pharmacology, University of Miami, Miami, FL USA; 2 Division of Surgical Oncology, DeWitt Daughtry Department of Surgery, University of Miami, Miami, FL USA; 3 Marine and Atmospheric Chemistry, Rosenstiel School of Marine and Atmospheric Science, University of Miami, Miami, FL USA; 4 Department of Pathology, University of Miami, Miami, FL USA; 5 Department of Molecular and Integrative Physiology, University of Michigan, Ann Arbor, MI USA

**Keywords:** Volatile organic compounds, Skin cancer, Metabolites, GCMS, Palmitic acid

## Abstract

The analysis of volatile organic compounds (VOC) as biomarkers of cancer is both promising and challenging. In this pilot study, we used an untargeted approach to compare volatile metabolomic signatures of melanoma and matched control non-neoplastic skin from the same patient. VOC from fresh (non-fixed) biopsied tissue were collected using the headspace solid phase micro extraction method (HS SPME) and analyzed by gas chromatography and mass spectrometry (GCMS). We applied the XCMS analysis platform and MetaboAnalyst software to reveal many differentially expressed metabolic features. Our analysis revealed increased levels of lauric acid (C12:0) and palmitic acid (C16:0) in melanoma. The identity of these compounds was confirmed by comparison with chemical standards. Increased levels of these fatty acids are likely to be a consequence of up-regulated de novo lipid synthesis, a known characteristic of cancer. Increased oxidative stress is likely to cause an additional increase in lauric acid. Implementation of this study design on larger number of cases will be necessary for the future metabolomics biomarker discovery applications.

**Electronic supplementary material:**

The online version of this article (doi:10.1007/s11306-013-0523-z) contains supplementary material, which is available to authorized users.

## Introduction

Cancer, as the leading cause of death worldwide, is projected to increase from 7.9 to 11.5 million, for a period from 2007 to 2030 (Thun et al. 2010). Early detection of cancer greatly increases the chances of successful treatment. If detected early (when confined to the primary site) the melanoma 5 year survival rate is about 98.2 % (Howlader et al. 2012). Unfortunately, the majority of melanoma cases are not detected in this early time window. Given its origin as a pigmented lesion on the skin, melanoma presents a unique opportunity for early detection. However detecting early melanoma in sun-damaged skin is not always an easy task, as the lesions may be masked by ephelides (freckles), seborrheic keratosis, pigmented actinic keratoses, lentigines or nevi (Moller et al. 2009). There have been several anecdotal reports of canine detection of melanoma (Williams and Pembroke 1989; Church and Williams 2001). Many studies have demonstrated the extreme olfactory ability of dogs to detect and distinguish cancer from non-cancer (Willis et al. 2004; McCulloch et al. 2006; Horvath et al. 2008; Sonoda et al. 2012). These observations suggest that the headspace over melanoma contains volatile molecules. The idea to use volatile organic compounds (VOC) or metabolites to aid in early detection of skin diseases and melanoma is not new (D’Amico et al. 2008; Gallagher et al. 2008; Abaffy et al. 2010). It has been demonstrated that metabolic changes due to a cancer process are reflected in the secretion products from exocrine glands and of volatiles released by the skin. Skin volatile compounds and their modifications caused by environmental factors have been studied using sorbtive tape extraction and in vivo sampling methods (Sisalli et al. 2006; Bicchi et al. 2007; Sgorbini et al. 2010).

In many different cancers, breath analysis was used to determine which volatile compounds-metabolites can serve as potential biomarkers. Phillips et al. studied volatile metabolites in lung and breast cancer patients (Phillips 1997; Phillips et al. 1999, 2003, 2010). Chen et al. (2007) used HS SPME GCMS method to extract volatile organic metabolites from breaths of lung cancer patients. In addition many other cancers have been investigated using chemical analysis of exhaled breath (ovarian, colon, prostate, liver and stomach cancers) (Peng et al. 2010; Meloni et al. 1999; Ligor et al. 2007; Sonoda et al. 2012). Using an animal model approach, Matsumura et al. showed that urine volatile metabolic signature could distinguish tumor-bearing mice from control animals (Matsumura et al. 2010). In addition, it has been demonstrated that the urine volatile metabolomic profile from leukemia, colorectal cancer and lymphoma can differentiate cancer patients from healthy individuals (Silva et al. 2011). Thus, VOC/metabolite analysis of skin as well as breath and urine can be a novel diagnostic approach to detect cancer.

Recent study tested the hypothesis that malignant melanoma tissue forms a unique volatile signature that is different than skin and nevi (non-neoplastic and benign lesional tissue) and demonstrated proof of principle that malignant melanoma tissue has a volatile profile distinct from healthy, non-neoplastic skin and nevi (Abaffy et al. 2010). In that study, mass spectra from a given sample were compared to the NIST reference library and the metabolites were identified if they had ≥60 % similarity. The major limitation of that study was the control tissue, which could not be matched for sex, age, environmental and other potential confounding factors. The aim of this preliminary study was to directly compare volatile metabolic profiles of melanoma (M) and non-neoplastic (non-melanoma, NM) skin using matched control, healthy, adjacent skin from the same patient, isolated and analyzed on the same day. We applied an untargeted approach, as this represents an efficient tool for early detection of disease (Ackermann et al. 2006; Sreekumar et al. 2009). We focused on validation of this novel approach, including spectra processing and statistical analysis. Due to the small sample size, this pilot study does not seek to identify biomarkers of melanoma. However, we did annotate several metabolic “features” that were significantly different between malignant melanoma and skin. Significant metabolites present in the melanoma were identified as fatty acids and confirmed with standards. Their presence may indicate increased cellular oxidative stress (C12:0) and increased cellular proliferation (C16:0 and C12:0) (Lindahl 1992; Rahman et al. 2002; DeBerardinis et al. 2008; Marchitti et al. 2008).

## Materials and methods

### Sample preparation and volatile collection

We obtained ethics approval from the Institutional Review Board (IRB). Biopsy samples were collected from subjects recruited in accordance with the approved IRB protocol (No. 2006117) after a written consent form was signed. Samples were subjected to HS SPME volatile collection and GCMS analysis as described previously (Abaffy et al. 2010, 2011). A total of ten patients were recruited: five with melanoma lesions (Table 1) and five with non-melanoma (benign lesions) (Table 2). Two tissue biopsies were performed on each patient with a 2 mm punch biopsy device, one from the lesion (melanoma or non-melanoma lesion) and the other from adjacent healthy skin from the same patient. The biopsy sample was placed in a vial (Agilent, No. 5182-0715, 1.5 mL with 0.3 mL polyspring insert, National Scientific, C4010-630, insert diameter 3 mm) and capped with a Teflon coated silicon septum, which sealed the air within the insert from the vial and the external environment. Tissue sample was placed at the bottom of the insert leaving approximately 15 mm of space above the tissue. The sample was kept on ice for a maximum of 2 h after biopsy and before the HS SPME volatile collection. Upon insertion of the SPME fiber, volatile compounds partition to the gaseous phase as well as to the SPME fiber coating. The extractive portion of the SPME fiber was 10 mm in length. It was completely exposed to the headspace above the tissue during the extraction period without directly contacting the sample. Under these circumstances, volatile compounds prefer to accumulate in the headspace, resulting in substantial loss of sensitivity when the headspace volume is too large. In order to minimize the headspace and to achieve higher sensitivity of headspace extraction, we placed a 0.3 mL insert into the vial. We collected volatile compounds using HS SPME device with 0.65 μm PDMS-DVB fiber for 1 h at room temperature. The manual HS SPME holder was inserted into injector of the GC (splitless mode) and volatiles from fiber desorbed (at 220 °C for 1 min). The SPME fiber was conditioned at 250 °C under a purified stream of helium prior to first use according to manufacturer’s instructions. Blank fiber was directly injected each day before the analysis. Only two samples (melanoma or non-melanoma and control healthy skin) were analyzed per day. We analyzed 20 tissues (five melanoma +5 matching skin and five non-melanoma +5 matching skin), and ten air samples. Analysis of air samples (volatile collection from the empty vial) was done in the same way as melanoma and skin samples.

**Table 1:** Demographics, diagnosis and histology analysis (H&E staining) of 5 matched melanoma cases

	Case M-1	Case M-2	Case M-3	Case M-4	Case M-5
Sex	M	M	M	M	F
Race	White	White	Hispanic dark skin	White	Hispanic dark skin
Age	49	73	35	75	38
Initial diagnosis	Nodular melanoma	Nodular nevoid melanoma	Acral lentigenous melanoma	Recurrent malignant melanoma	Melanoma
Localization	Right forearm	Right temple	Right foot	Scalp	Back
Tissue/lesion	Intact	Previously shaved	Intact	Previously shaved	Intact
Breslow thickness	0.98 mm	0.75 mm	2.2 mm	0.67 mm	5 mm
Clark level, TNM stage	II, T1N1M0	IV, T1N0M0	V, T3aN2M0	T1bN0M0	V, T4bN1M0
Biopsy sites					
Diagnosis from our punch biopsy sample	Malignant melanoma	Melanoma in situ	Acral lentigenous melanoma	Melanoma in situ	Melanoma
Histology-melanoma					
Histology-adjacent, non-neoplastic skin					

The site of punch biopsy of melanoma (indicated with  ) and of adjacent skin are indicated with white circle. Unfortunately, we did not obtain the macroscopic picture for case M-5

**Table 2:** Demographic, diagnosis and histology analysis of five non-melanoma cases

	Case NM-1	Case NM-2	Case NM-3	Case NM-4	Case NM-5
Sex	M	M	M	M	F
Race	White	White	White	White	White
Age	50	83	64	34	77
Initial diagnosis	Melanoma	Lentigo maligna melanoma	Melanoma in situ	Melanoma in situ	Melanoma in situ
Localization	Vertex of the scalp	Left temple	Left cheek	Right posterior auricular	Forehead
Tissue/lesion	Previously shaved	Intact	Previously shaved	Previously shaved	Previously shaved
Breslow thickness	0.82 mm	1.1 mm	–	–	–
Clark level, TNM stage	I, T1N1M0	V, T2aN0M0	I, TisN0M0	I, TisN0M0	I, TisN0M0
Biopsy sites					
Diagnosis from our punch biopsy sample	No residual melanoma	No residual melanoma	No residual melanoma	No residual melanoma	No residual melanoma
Histology-non-melanoma					
Histology-adjacent, non-neoplastic skin					

The site of punch biopsy of non-melanoma (indicated with  ) and of nearby skin are indicated with white circle. The macroscopic picture for Case NM-5 is missing, because initial diagnosis was done in the primary care office

### GCMS analysis

GSMS analysis was performed using an HP 6890 gas chromatograph and Agilent 5973 quadrupole mass spectrometer operating in the electron impact (EI) mode. The filament off time was 0.1 min. This was used to reduce the signal from gases N_2_ and CO_2_ that are injected via the SPME fiber. Before each analysis set the instrument was tuned via the autotune function of the instrument. Our program was: 40 °C for 2 min followed by 6 °C/min ramp to 270 °C and hold for 5 min. Helium carrier gas flow was set at 0.7 mL/min. Agilent 5973 mass spectrometer was used in the full scan mode at a mass range of 30–300 amu. Column used: DB-5MS, model J&W 128-5522, 25 m × 0.2 mm I.D. × 0.33 μm film. EI ionization used was 70 eV. DB-5MS is a phenyl arylene polymer equivalent to a 5 %-phenyl-methylpolysiloxane (DB-5). Reproducibility of the method was tested using lauric acid as a standard. The reproducibility or precision was determined as % of relative standard deviation (%RSD) and was 8.6 % (*n* = 7).

### Chemometric data analysis

After GCMS analysis, raw data were converted to netCDF files using MASSTransit software (by Palisade-Scientific Instrument Services, Ringoes, NJ, USA). GCMS spectra processing were carried out using freely available XCMS software (online version 1.21.01, The Scripps Center for Metabolomics, La Jolla, CA, USA) (Smith et al. 2006) using the default parameters for GC Single Quadruple [general: GC/single quad MS (matched Filter), GC-EI, single quadruple MS, retention time (RT) format: minutes, polarity: positive, feature detection: matched Filter, step: 0.25, FWHM: 3, RT correction: method: peak groups, non-linear/linear alignment: loess, extra: 1, missing: 1 Alignment: mzwid: 0.25, minfrac: 0.5, bw: 10). GCMS spectra processing included: filtering and identifying peaks, matching peaks across samples, peak RT alignment and arranging peaks into peak intensity table for further statistical analysis]. Thus, all chromatograms were simultaneously analyzed with identical settings. The processed data were uploaded into freely available MetaboAnalyst software for statistical analysis (www.metaboanalyst.ca) (Xia et al. 2009; Xia and Wishart 2011). The total ion count (TIC) for each chromatogram varied between the samples, as seen in Supplementary Table ST1. The source of this variation is likely to be biological, (different skin type and localization on the body). In addition, some of the variation may come from differences in the instrumental conditions. Although analytical conditions were controlled, samples were collected and analyzed over several months and thus slight changes in the SPME fiber properties over time may have contributed to the variation seen in TIC. On the other hand, the change in TIC in each pair sample (melanoma vs. skin and non-melanoma vs. skin) was negligible and we used pairwise analysis throughout our manuscript to investigate differences between these two groups. The intensity signal of each metabolic feature is expressed in relative terms, as a percentage of TIC/intensity signals of the feature in each sample. For the untargeted approach, the entire chromatogram (all *m*/*z* values) is equally important. In a complex sample like skin, accurate compound identification is challenging. Some peaks may contain mixtures of metabolites that are co-eluting, so the individual MS scans of their peaks are not pure spectra of either metabolite, thus making it difficult to achieve high spectral matching with the reference library. Some chromatographic peaks with lower abundance are difficult to distinguish from the noise. To overcome these problems we used a chemometric approach, meaning that the chemical compounds were not annotated before the statistical analysis, only their mass spectra (mass to charge ratio, *m*/*z* value) and the RT of the chromatographic peak (RT) were recorded as a metabolic “feature”. Since individual chemical compounds give rise to more than one fragment ion upon ionization, these ion-features, generated by XCMS, were grouped together using the CAMERA algorithm (Smith et al. 2006; Kuhl et al. 2012). Annotation of a given mass spectrum was assigned only after a metabolic “feature” showed a statistically significant differential profile using Student t-tests and fold change analysis. The Chemical Analysis Working Group of the Metabolomics Standards Initiative (MSI, http://msi-workgroupssourceforge.net) (Sumner et al. 2007) has defined four different levels of metabolites identification confidence. The identity of lauric and palmitic acid was confirmed with level 1 confidence with Sigma Aldrich W261408 and P0500 standards, respectively. In addition, retention indices were used in combination with mass spectral analysis (similarity search) to help with annotation of four additional volatiles. For these purposes a Restek standard (Restek, Bellefonte, PA, USA) containing 50 μg/mL of n-alkane mix (Cat. No. 31633) was run using the same instrument parameters and experimental conditions as for melanoma samples. RTs of each hydrocarbon standard eluted was recorded and the RI calculated as described in (Lucero et al. 2009).

## Results

**Fig. 1:**
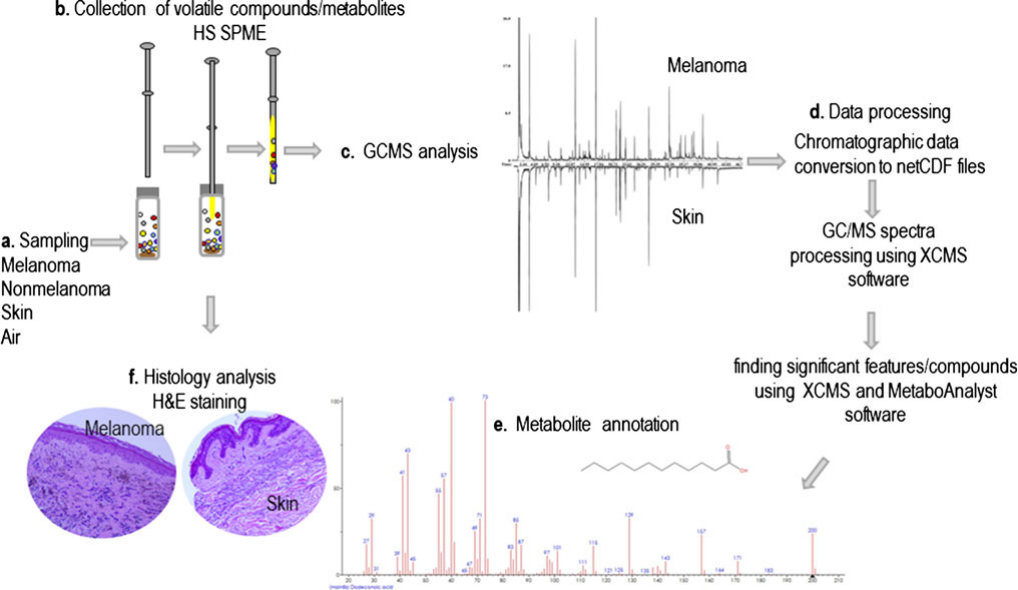
Study design. *a* Tissue sampling. *b* Collection of volatile compounds using head space solid phase micro-extraction method (HS SPME). *c* Gas chromatography–mass spectrometry analysis (GCMS). *d* Data processing includes alignments of all chromatograms, baseline correction, filtering and statistical analysis. *e* Metabolite annotation. *f* Histology analysis, pathology status confirmation

Our study design is presented in Fig. 1 and a detailed description of each step is given in “Materials and methods” section. Here, we describe the comparison of volatile metabolomic signatures from three groups: air versus skin samples, melanoma versus matching skin (Table 1) and non-melanoma versus matching skin (Table 2).

### Comparison of skin and air samples

**Fig. 2:**
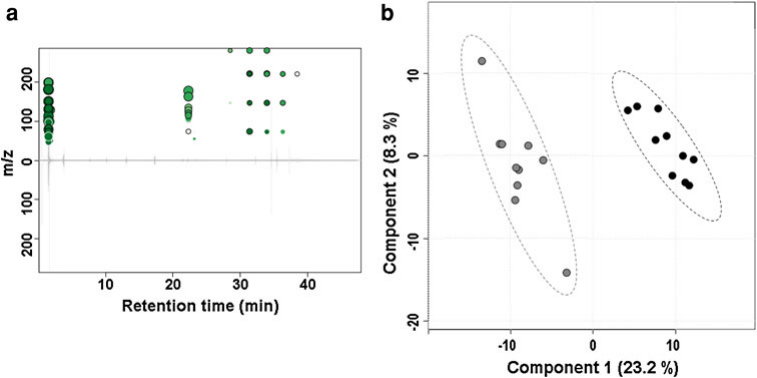
XCMS analysis. **a** Mirror plot. Mirror plot showing up-regulated metabolic features-ions (*green circles*) altered between skin and air samples. Note, that there are no down-regulated features. The size of each *circle* corresponds to the (log) fold change of the feature (the average difference in relative intensity of the peak between sample groups). Thus, *larger circles* correspond to peaks with greater fold change. Also, intensity of the *color* is used to represent *p* value with *brighter circles* having lower *p*-values, i.e. higher significant difference (Tautenhahn et al. 2012). **b** PLS DA score plot. A clear separation between the air (*grey circles*) and the skin (*black circles*) group (% of variance for each principal component is presented too) (Color figure online)

We analyzed ten air against ten skin samples (five from melanoma cases and five from non-melanoma cases). Volatile collection in these samples was not matched, meaning samples from these two groups were not collected at the same time. Chromatographic data were processed as described in “Materials and methods” section. The results from XCMS analysis, a mirror plot, summarizing most significant up-regulated features-ions whose intensities were altered between skin and air samples using thresholds of *p* ≤ 0.01 and fold change ≥1.5 is presented in Fig. 2a. A total of 77 metabolic features were identified. The table of absolute intensity peaks from XCMS was transformed to the relative intensities table in each particular sample and imported into MetaboAnalyst for the statistical analysis (matrix of 20 samples and 400 features). Data were normalized using the autoscaling option. Three hundred and nine features with ≥1.5 fold change in relative expression value were identified when air and skin group was compared. There were 158 features identified as significant by paired t-tests with *p* value ≤0.05. Data were clearly separated in two groups using unsupervised Principal Component Analysis (PCA) analysis (Supplementary Fig. 1). In addition, we performed a partial least squares-discriminant analysis (PLS-DA). Figure 2b shows the representative points of the air and skin samples mapped in space spanned by the first two components in the PLS-DA. There is a clear separation between air (grey circles) and skin (black circles) group. The PLS-DA plot suggests that 23.2 % of the variance is the difference between air and biopsy tissue. It does appear that the difference between air and biopsy tissue does not capture the majority of the variance in the data. However, all of the remaining variance is not necessarily independent of this difference. For example, we have only shown the two PLS components which best achieve a compromise between two aims, namely, describing or capturing the explanatory variables and predicting the response or tissue class. There may be other PLS components that explain the variance which separates the air and biopsy data sets, but may be less predictively useful. Also, PLS-DA likely does not capture all of the variability between air and biopsy tissue. It is a technique which tries to find the best lower dimensional projections of the data based on certain assumptions about the data (normality, linearity). It is likely that such complex data does not meet these assumptions, so PLS-DA is likely to only capture a portion of the overall variability.

### Comparison of melanoma cases versus matching skin controls

First, we report the analysis of volatile metabolic profiles from five confirmed melanoma patients using fresh skin tissue biopsy from melanoma lesion and from uninvolved adjacent skin from the same patient. Detailed demographic data, diagnosis, localization, punch biopsy sites, final pathology report and histology pictures are presented in Table 1. Melanoma tissues from cases M-1, M-3 and M-5 were not subjected to shave biopsy prior to punch biopsy for the current study (intact). Pre-surgical diagnoses in these cases were obtained from other lesions on the skin and confirmed histologically after volatile analysis. We obtained paired melanoma and uninvolved adjacent skin samples and analyzed them on the same day. We analyzed results using relative intensity values. The relative intensity signal was defined as the absolute signal intensity value divided by the TIC from each sample and imported into MetaboAnalyst software. This approach normalizes the TIC from each collection to control for variations in collection efficiency with the SPME fiber and the GC column. Previous studies reporting GCMS analysis have listed a huge number of features, but identification of only a small number of compounds (Bottcher et al. 2008). Thus, an additional step to categorize these features into smaller feature groups (FG) was applied. Each FG contains features that are likely related to the same compound based on RT, peak shape and intensity correlations (Kuhl et al. 2012; Tautenhahn et al. 2012). Our 487 features were thus classified into 105 FG. In MetaboAnalyst, data were normalized using the autoscaling option. Six significant features, assigned to six FG, were identified by *t* test assuming equal variances and are listed in the Supplementary Fig. S2a. Important features identified by fold change (≥1.5X) analysis were presented in Supplementary Fig. S2b.

### Annotation of significant feature groups

Next, we wanted to annotate these six FG (FG: 23, 36, 54, 56, 60 and 79, Table 3). We manually analyzed peaks at the given RT and compared these mass spectra with the library hits from NIST database. In addition, retention index was calculated, as described in “Materials and methods” section, to help in annotation of mass spectra. The annotations of the FG together with their retention indices are listed in Table 3. The annotated compounds found to be significantly increased in melanoma group were 2-ethylhexyl-4-methoxycinnamate, 1-eicosene; lauric; palmitic; and myristic acid. Only one compound, toluene, was identified as decreased in melanoma relative to skin. Since the medication used by our melanoma patients was not uniform Supplementary Table ST2) and because these compounds were identified relative to the paired healthy skin control biopsy from the same patient, we are confident that none of the volatile compounds listed in the Table 3 are secondary metabolites of medication. Of these compounds, cinnamaldehydes are known to be present in cosmetic products and also in our skin (Gallagher et al. 2008). At this point, it is difficult to be certain about its origin in our melanoma samples.

**Table 3:** Annotation of significant features using retention index (RI) and spectral similarity search with NIST 08 v.2.0 library

Feature group	Features	RT (min)	RT (sec)	Calculated RI	Estimated RI from NIST 08 v.2.0 (iu)	NIST match factor	Name	Change
23	M290T39	39.08	2,345	2,208	2,088	822	2-Ethylhexyl-4-methoxy-cinnamate	**+**
36	M56T37	37.23	2,234	2,112	1,997	846	1-Eicosene	**+**
54	M60T26, M73T26, M60T26	26.35	1,581	1,565	1,570	**916**	**Dodecanoic or lauric acid**	**+**
56	M55T34, M57T34, M60T34, M73T34, M256T34	33.50	2,010	1,964	1,968	**864**	**Hexadecanoic or palmitic acid**	**+**
60	M91T6, M92T6	5.80	348	749	758*	899	Toluene	–
79	M60T30	30.08	1,805	1,763	1,769	871	Tetradecanoic or myristic acid	**+**

The last column indicates the direction of change in melanoma group

* RI value for toluene from NIST library, using a capillary, standard non-polar, DB-1 column

Bold entries indicate statistical significance (*p* < 0.05)

**Fig. 3:**
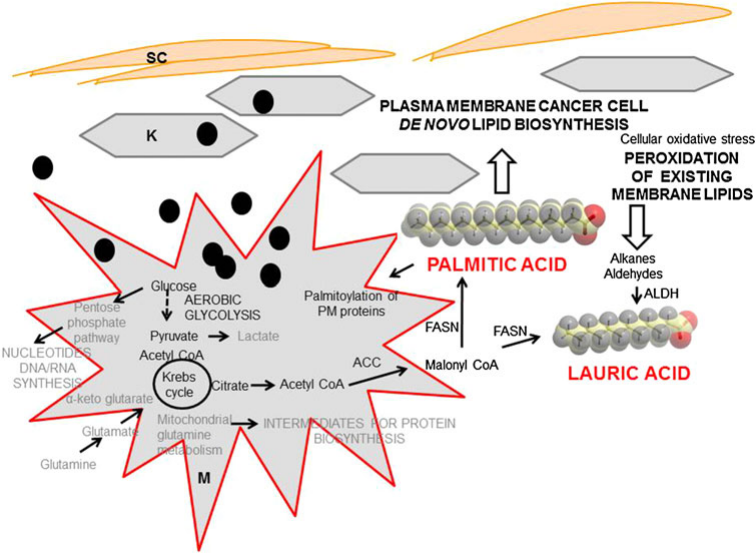
Palmitic and lauric acid as major volatiles implicated in metabolic changes of melanoma cell proliferation. *SC* stratum corneum, *K* keratinocyte, *M* melanoma cell, *ACC* acyl CoA carboxylase, *FASN* fatty acid synthase, *ALDH* aldehyde dehydrogenase, *PM* plasma membrane, *black circle* melanosome

### Comparison of five non-melanoma cases versus matching skin controls

We also analyzed volatile profiles of five non-melanoma cases, which had been initially diagnosed as melanoma based on previous shave-biopsy histological analysis. After diagnosis based on the original shave-biopsy, patients were scheduled for surgical excision of the lesion. In these five surgical excision biopsy samples the absence of residual melanoma was confirmed histologically (Table 2). XCMS analysis of the raw data was performed and there were no dysregulated features detected between non-melanoma and matching skin, using 1.2 fold change threshold and using *p* value significant threshold of 0.09 and highly significant threshold of 0.05. It is important to mention that the inability to find differentially expressed features between non-melanoma and adjacent control matched skin (Table 2) is in agreement with histologic analysis which did not find neoplastic changes (consistent with the absence of melanoma).

## Discussion

Our primary interest, due to a small sample size, was to present the improved methodological control for the potential confounding factors: non-neoplastic, adjacent skin from the same patient. We believe that this is a significant improvement in study design. Following stringent criteria for compound identification based on proposed minimum reporting standards for chemical analysis from the MSI (Sumner et al. 2007), we annotated volatile compounds based on their spectral similarity with library match and retention index. Six FG with statistically significant differences between histologically confirmed melanoma and adjacent non-neoplastic skin obtained from the same patient during a single surgical session were listed in Table 3. The identities of lauric and palmitic acid were confirmed at level 1 confidence using chemical standards and according to the MSI criteria. Human Metabolome Database search (www.hmdb.ca) lists palmitic acid (HMDB00220) as one of the most common saturated fatty acids found in animals and plants, with detectable concentrations in human serum, cerebrospinal fluid and urine. Lauric acid (HMDB00638) was also found in human serum, cerebrospinal fluid and urine. Our analysis indicates significantly increased levels in melanoma relative to adjacent uninvolved skin.

VOC released from skin form body odor and convey important information about our metabolism (Bernier et al. 2000; Gallagher et al. 2008). It is well known that volatile profile of human skin changes with age, nutritional status and environmental factors. At older age, decreased hormonal levels result in decreased secretion from apocrine and sebaceous glands and ultimately lead to change in volatile profile and skin odor (Zouboulis et al. 2007; Chen et al. 2007). In addition to the topographic and age related variations in the lipid composition of human skin, variations in lipids and their secondary metabolites due to cancerous process are also present (Abaffy et al. 2010). Thus, our adjacent non-neoplastic skin is the best choice of control.

What about nevi? A differential volatile profile in nevi when compared to melanoma has been shown,, however these samples were not matched (Abaffy et al. 2010). Increased number of nevi and extent of freckling is an important risk factor for melanoma (Tucker 2009). Results from clinical and histological data indicate that about 25 % of melanoma arise from or are associated with pre-existing nevus (Marks et al. 1990; Tsao et al. 2003). Nevi are considered a benign tumor of melanocytes that have oncogene-induced senescent phenotype (non-proliferating cells) (Michaloglou et al. 2005). This is a barrier to malignant transformation. However, it has also been shown that some markers of senescence are expressed in melanoma (Reed et al. 1995; Tran et al. 2012). Together, these data call for caution when comparing melanoma and nevi, and add an additional level of complexity if one was to consider nevi as a proper control.

The question about the biochemical origin of these metabolites arises from our results. It is known that during early stages of melanoma progression, innate and adaptive immune cells are recruited to the tumor site (e.g. macrophages). In addition, structural components of extracellular matrix including collagens, elastins and soluble factors like cytokines, chemokines and polypeptide growth factors are released either from neoplastic or non-neoplastic cells. It is known that tumor associated macrophages release matrix metalloproteinase (MMP) and elastase and are thus actively involved in breaking and remodeling of extracellular matrix (Schmid and Varner 2010). Two possible not mutually exclusive explanations related to the origin of palmitic acid exist: (1) the necessity for the increased lipid synthesis due to cell growth and proliferation in cancer and (2) the endogenous presence of C16 in the skin is more prominent in melanoma skin lesion due to changes in Stratum Corneum and melanoma cell microenvironment. First, increased de novo fatty acid (FA) biosynthesis is a very important metabolic alteration associated with cell proliferation and cancer (DeBerardinis et al. 2008). Fatty acid synthase over-expression in melanoma has been correlated with Breslow thickness and overall poor survival (Innocenzi et al. 2003; Kapur et al. 2005; Byrum et al. 2011). It is highly likely that our increased pool of palmitic acid serve as a building block for plasmalogenes synthesis (Fig. 3). Plasmalogens are phospholipids with vinyl ether linkage (Han and Gross 2005). As a general rule, the amounts of the plasmalogens in the plasma membrane do not exceed more than 1 %. However, if they amount for >4 % of total phospholipids it correlates with neoplastic transformation (Mangold and Paltauf 1983; Smith et al. 2008). High temperature GCMS have been used to study skin surface lipids and predominant fatty acid identified were hexadecenoic or sapienic acid (C16:1) and hexadecanoic or palmitic acid (C16:0) (Michael-Jubeli et al. 2011). This poses a question as to whether or not a disruption of Stratum Corneum integrity due to melanoma results in increased palmitic acid volatile profile. If so, it could represent an additional and/or alternative source of increased palmitic acid pool.

Our analysis also reveals increased lauric acid in melanoma. This medium-chain fatty acid can originate from increased de novo fatty acids biosynthesis present in cancer, but it can also be a product of increased aldehyde dehydrogenase activity (ALDH) reported to be present in many cancers (Lindahl 1992). Elegant work by Boonyaratanakornkit et al. (2010) showed that melanoma initiating cells expressing high ALDH activity develop xenograft melanomas with high proliferative and self-renewal ability. Increased lipid peroxidation can also be an indirect source of aldehydes present in cancer cell. Aldehydes derived from the lipid peroxidation are oxidized to carboxylic acids by ALDH, and this is in most cases considered as a detoxification process (Vasiliou et al. 2000). Thus, lauric acid present in our melanoma group suggests that it may be due to increased plasma membrane lipid peroxidation.

The only volatile compound found to be decreased in the melanoma group is toluene. It is tempting to speculate that it is an environmental contaminant and not an endogenous metabolite; however, a few studies based on much larger sample size have also identified toluene as a discriminant compound identified as decreased in the breath of lung cancer non-smoker patients (Rudnicka et al. 2012).

## Conclusions

The use of volatile organic metabolites as biomarkers of cancer is a new frontier in cancer research. Our analysis revealed increased levels of lauric acid and palmitic acid in melanoma. While these results are encouraging, a larger effort will be required to fully validate these findings and assign these volatiles as biomarkers of melanoma. Increased levels of these fatty acids are likely to be a consequence of up-regulated de novo lipid synthesis and increased oxidative stress. We believe that our approach of collecting volatile organic metabolites in the headspace above the cancer tissue has a future potential to become a non-invasive in situ collection (without a biopsy). This will enormously speed up biomarkers research discovery and validation. Implementation of this study design on larger number of cases will be necessary for the future metabolomics biomarker discovery applications.

## Electronic supplementary material

Below is the link to the electronic supplementary material.

Supplementary material 1 (TIFF 126 kb)
Unsupervised PCA analysis of air and skin samples (air = red, green = skin)

Supplementary material 2 (TIFF 8076 kb)
Significant features. a. Significant features identified by paired *t*-test between melanoma and matching skin samples. b. Fold change analysis. Box-Whisker plot summary of the each significant features identified by paired FC analysis using ≥1.5 FC threshold (S = control, skin, grey, M = melanoma, red)

Supplementary material 3 (DOCX 14 kb)

Supplementary material 4 (DOCX 13 kb)
