# Supplementary figures and images for: Comparative analysis of volatile metabolomics signals from melanoma and benign skin: a pilot study
Source: Metabolomics. 2013 Mar 30;9(5):998–1008. doi: 10.1007/s11306-013-0523-z (PMC3769583; doi:10.1007/s11306-013-0523-z)

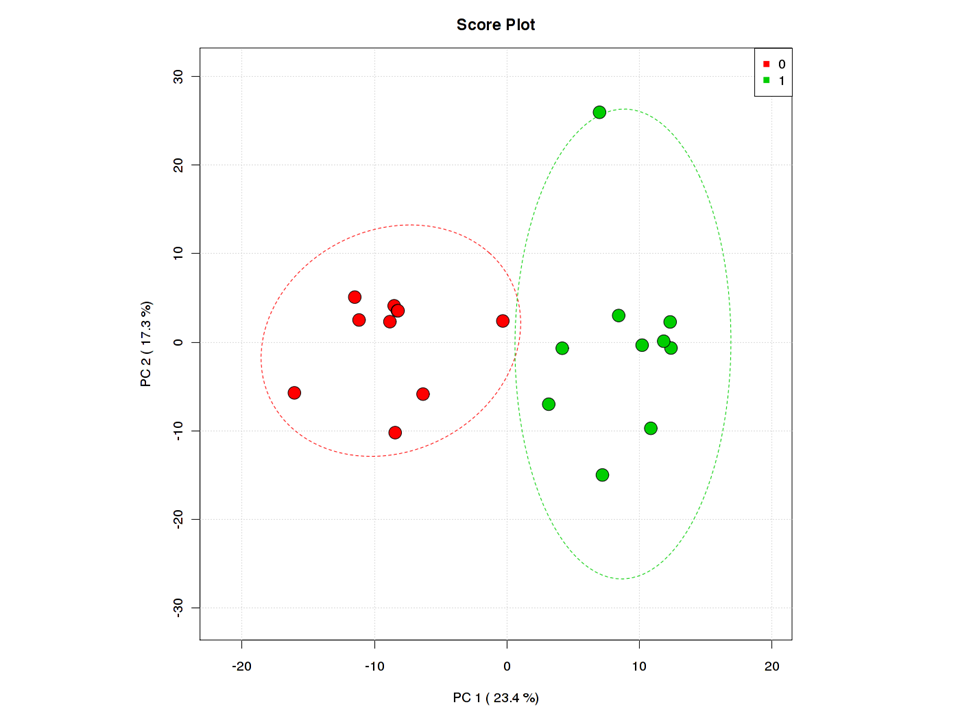

Supplement: Supplementary file 1 — Supplementary material 1 (TIFF 126 kb) Unsupervised PCA analysis of air and skin samples (air = red, green = skin) [file 11306_2013_523_MOESM1_ESM.tif]

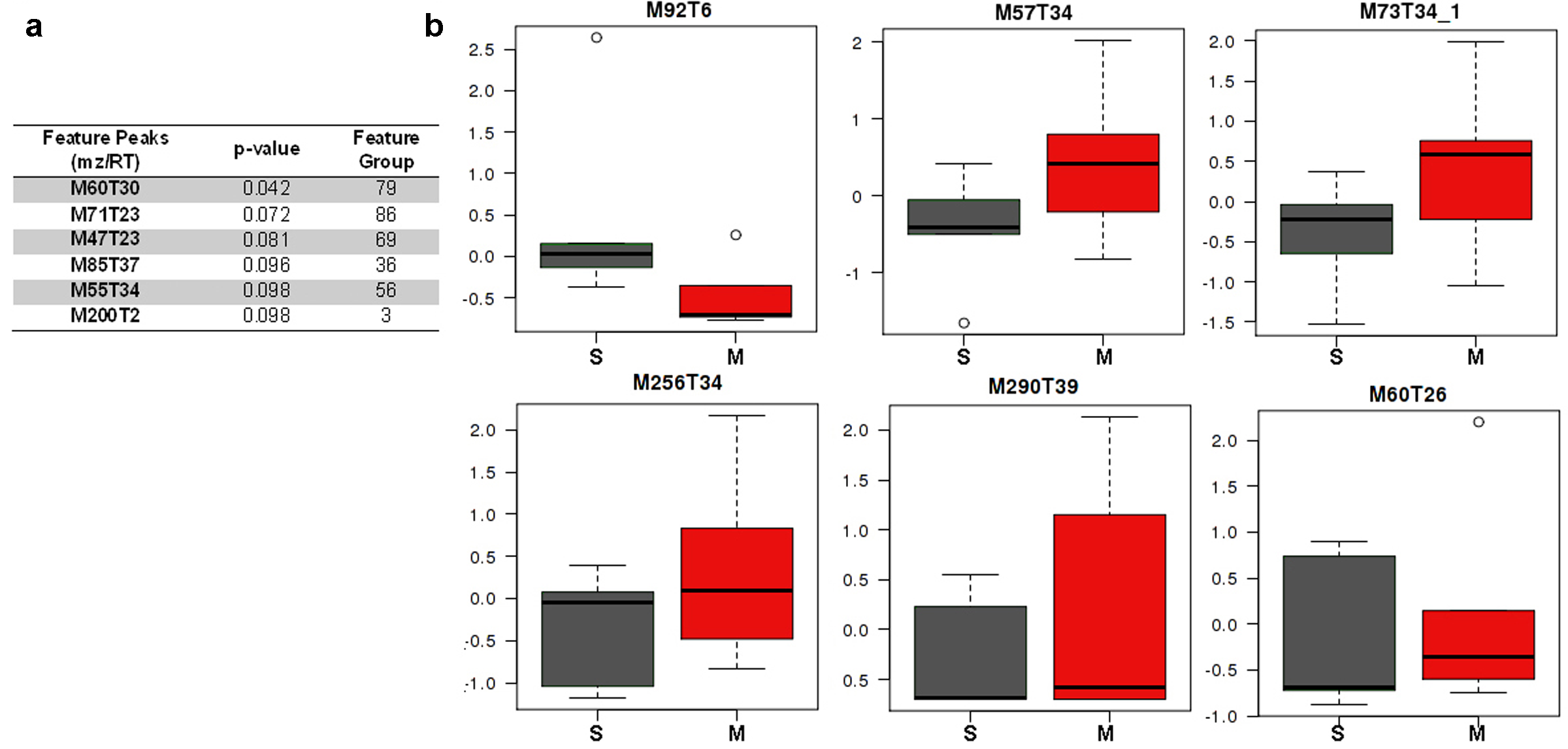

Supplement: Supplementary file 2 — Supplementary material 2 (TIFF 8076 kb) Significant features. a. Significant features identified by paired t-test between melanoma and matching skin samples. b. Fold change analysis. Box-Whisker plot summary of the each significant features identified by paired FC analysis using ≥1.5 FC threshold (S = control, skin, grey, M = melanoma, red) [file 11306_2013_523_MOESM2_ESM.tif]
