# Supplementary material for: Comparative analysis of volatile metabolomics signals from melanoma and benign skin: a pilot study
Source: Metabolomics. 2013 Mar 30;9(5):998–1008. doi: 10.1007/s11306-013-0523-z (PMC3769583; doi:10.1007/s11306-013-0523-z)
Supplement: Supplementary file 3 — Supplementary material 3 (DOCX 14 kb) [file 11306_2013_523_MOESM3_ESM.docx]

**Supplementary Table ST1. TIC-Total Ion Count.** (*matching samples- melanoma or non-melanoma samples and control skin samples were from the same patient. Biopsy and the volatile collection were performed on the same day).

| **Sample No**  **Control skin*** | **TIC** | **Sample No**  **Melanoma*/Non-melanoma*** | **TIC** | **Sample No**  **AIR** | **TIC** |
| --- | --- | --- | --- | --- | --- |
| **1** | 63196827 | M-1 | 69884322 | A-1 | 2379895 |
| **2** | 32807429 | M-2 | 29559361 | A-2 | 1232191 |
| **3** | 15953084 | M-3 | 24716585 | A-3 | 2382777 |
| **4** | 15874458 | M-4 | 21685103 | A-4 | 2539534 |
| **5** | 28445664 | M-5 | 26054381 | A-5 | 2061447 |
| **6** | 4984144 | NM-1 | 3136333 | A-6 | 1990792 |
| **7** | 26473862 | NM-2 | 28853770 | A-7 | 2767273 |
| **8** | 28115550 | NM-3 | 46521769 | A-8 | 9966513 |
| **9** | 27316812 | NM-4 | 39007860 | A-9 | 21180500 |
| **10** | 8672351 | NM-5 | 9444944 | A-10 | 11072894 |
