# Supplementary material for: Comparative analysis of volatile metabolomics signals from melanoma and benign skin: a pilot study
Source: Metabolomics. 2013 Mar 30;9(5):998–1008. doi: 10.1007/s11306-013-0523-z (PMC3769583; doi:10.1007/s11306-013-0523-z)
Supplement: Supplementary file 4 — Supplementary material 4 (DOCX 13 kb) [file 11306_2013_523_MOESM4_ESM.docx]

**Supplementary Table ST2. List of medications used by melanoma patients during study.**

| **Case No** | **Medications** |
| --- | --- |
| Case M-1 | Phenytoin, Lisinopril, Amlodipine, Rosuvastatin, Ezetimibe, Celecoxib |
| Case M-2 | Ramipril, Mesalamine, Atorvastatin, Atenolol, Aspirin, Omeprazole |
| Case M-3 | None |
| Case M-4 | Olmesartan-hydrochlorothiazide |
| Case M-5 | Acetaminophen |
